# Generative priors for MRI reconstruction trained from magnitude-only images using phase augmentation

**DOI:** 10.1098/rsta.2024.0323

**Published:** 2025-06-19

**Authors:** Guanxiong Luo, Xiaoqing Wang, Moritz Blumenthal, Martin Schilling, Raviteja Kotikalapudi, Erik Rauf, Niels Focke, Martin Uecker

**Affiliations:** ^1^ University Medical Center Göttingen, Gottingen, Germany; ^2^ German Centre for Cardiovascular Research (DZHK), Lower Saxony partner site, Göttingen, Germany; ^3^ Athinoula A Martinos Center for Biomedical Imaging, Massachusetts General Hospital, Charlestown, MA, USA; ^4^ Institute for Diagnostic and Interventional Radiology, University Medical Center Göttingen, Gottingen, Niedersachsen, Germany; ^5^ Institute of Biomedical Imaging, Graz University of Technology, Graz, Steiermark, Austria; ^6^ Clinic for Neurology, University Medical Center Göttingen, Gottingen, Niedersachsen, Germany

**Keywords:** magnetic resonance imaging, image priors, inverse problem, image reconstruction, parallel imaging, regularization

## Abstract

In this work, we present a workflow to construct generic and robust generative image priors from magnitude-only images. The priors can then be used for regularization in reconstruction to improve image quality. The workflow begins with the preparation of training datasets from magnitude-only magnetic resonance (MR) images. This dataset is then augmented with phase information and used to train generative priors of complex images. Finally, trained priors are evaluated using both linear and nonlinear reconstruction for compressed sensing parallel imaging with various undersampling schemes. The results of our experiments demonstrate that priors trained on complex images outperform priors trained only on magnitude images. In addition, a prior trained on a larger dataset exhibits higher robustness. Finally, we show that the generative priors are superior to $\ell^{1}$ -wavelet regularization for compressed sensing parallel imaging with high undersampling. These findings stress the importance of incorporating phase information and leveraging large datasets to raise the performance and reliability of the generative priors for MR imaging (MRI) reconstruction. Phase augmentation makes it possible to use existing image databases for training.

This article is part of the theme issue ‘Generative modelling meets Bayesian inference: a new paradigm for inverse problems’.

## 1. Introduction

Magnetic resonance imaging (MRI) is a widely used non-invasive technique, but a key challenge lies in balancing imaging speed with image quality. This compromise is primarily determined using k-space measurements, which trace defined trajectories in the spatial Fourier domain. Regularizing the inverse problem for parallel MRI reconstruction is an effective and flexible approach for improving image quality, especially when the obtained k-space is highly undersampled to shorten the scan time. The prior knowledge that images are sparse in a transform domain as used in compressed sensing is known as $\ell^{1}$ -norm regularization [1,2]. Combined with incoherent sampling, this allows recovery of images from moderately undersampled k-space data with clinically acceptable quality [3,4].

The application of deep learning makes it possible to further increase undersampling without compromising image quality by leveraging the learned prior information from a training dataset. Popular methods can be classified into three main categories: supervised methods [5,6], where the neural network is a result of unrolling an iterative algorithm trained with labels and used to predict the reconstruction, self-supervised methods [7,8] that involve splitting the acquired k-space data of a scan into two disjoint sets, where only the first set is used for reconstruction and the second set provides supervision and decoupled methods [9–11], where a generative model or a denoiser is trained to learn the empirical distribution of data, which is then used in a conventional iterative reconstruction method. In the following, we will refer to a generative model also as a prior.

Training in supervised methods based on unrolled iterative algorithms requires not only fully sampled k-space data but also pre-defined sampling patterns and precomputed coil sensitivities. The prior knowledge learned in this way then pertains to these pre-defined settings. However, protocol settings for clinical research are changed often, and the preparation of reference data, used as labels for training, is costly. Decoupled methods are able to avoid these constraints, and the learned prior can even be transferred to new scenarios such as different contrasts [12].

As a crucial part of decoupled methods, the use of generative models, such as variational autoencoders and autoregressive models, was investigated previously by formulating the linear reconstruction problem for accelerated MRI from the Bayesian perspective and solving it via maximum *a posteriori* estimation [9,11]. More recently, diffusion models emerged as effective priors for MRI reconstruction and were combined with Monte Carlo methods sampling the posterior [12–14]. However, their performance is heavily dependent on the size and quality of the training dataset and the computational resources available.

**Figure 1.**
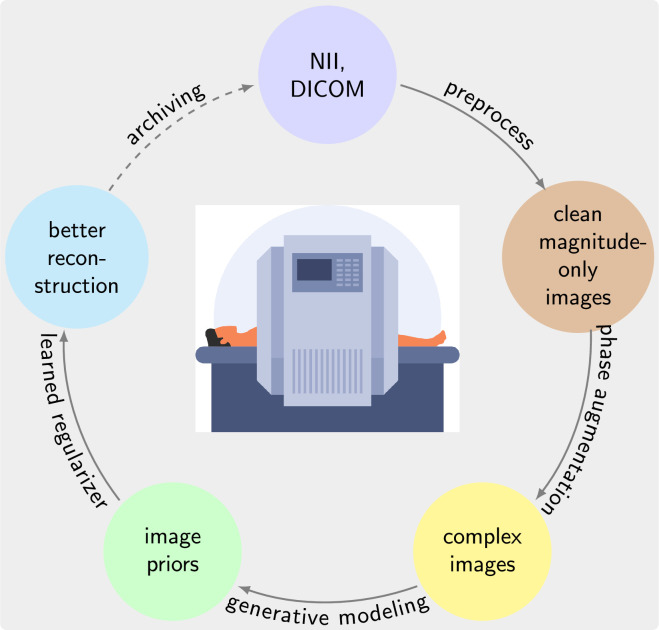
The proposed workflow for extracting prior knowledge and using it for regularization in image reconstruction. It comprises data preparation, phase augmentation, generative modelling and concludes with the use as learned regularizers in reconstruction.

For this reason, it is desirable to use existing databases of magnetic resonance (MR) images for training. But as shown here, training from magnitude-only images leads to inferior priors. This work therefore proposes a new approach to construct priors using magnitude-only training images as illustrated in figure 1. The workflow begins with the preparation of training datasets from magnitude-only MR images. This dataset is then augmented with phase information and used to train generative priors on complex images. Finally, trained priors can be used with both linear and nonlinear reconstruction for compressed sensing parallel imaging. The contributions of our work are:

**Complex versus magnitude-only priors.** We demonstrate that priors trained on complex images are superior to priors trained only on magnitude images.

**Phase augmentation.** We leverage a diffusion model trained on a small dataset (approx. 1000 images) of complex images to augment a much larger dataset (approx. 80 000 images) for which the phase information of the image is not available.

**Robustness.** We show that we can train more robust generative priors by incorporating knowledge from a larger training dataset, which contains a diverse range of images. Furthermore, the robustness is verified in different reconstruction scenarios that involve different sampling patterns, changes in echo time and repetition time (TE and TR, respectively), different scanners and so on. Such a database can be obtained by phase augmentation of magnitude images that are readily available.

**Flexibility.** By integrating the priors as regularization terms into existing reconstruction techniques, we maintain the flexibility of existing reconstruction algorithms (linear and nonlinear) that can be used with various undersampling schemes and receive coils.

Parts of this work have been presented in [15,16].

## 2. Theory

### (a) Linear and nonlinear reconstruction

The reconstruction in parallel imaging can be formulated as an inverse problem,


(2.1) $$F(x,c):=(F_{S}(x⊙c_{1}),\cdots ,F_{S}(x⊙c_{nc}))=y,$$

where $F_{S}$ is an undersampled multi-channel Fourier transform operator and the correspondingly obtained k-space data are $y=(y_{1},\cdots \;,y_{nc}{)}^{T}$ ; $y\in {\mathbb{C}}^{d\times nc}$ , $x\in {\mathbb{C}}^{n\times n}$ denote the image content and $c=(c_{1},\cdots \;,c_{nc}{)}^{T}$ ; $c\in {\mathbb{C}}^{n\times n\times nc}$ denote the coil sensitivities. Here, d is the number of samples in k-space, nc is the number of coils and n×n is the image size. Equation (2.1) can be solved in the following two ways.

One common way for MR image reconstruction is to predetermine the coil sensitivities c from a reference scan or from a fully sampled k-space centre. Following coil estimation, we can solve the linear inverse problem using the optimization regularized least-squares functional


(2.2) $$\begin{array}{lcr} & \underset{x}{\min\;}\frac{1}{2}\|F_{c}(x)-y{\|}^{2}+\alpha R(x), & \end{array}$$

where $F_{c}(x):=F(x,c)$ is a linear operator and R(x) is the regularization term employing prior knowledge about the image, such as $\ell^{2}$ -regularization [17], total variation [2], $\ell^{1}$ -sparsity [1] or a learned log-likelihood function [11].

Alternatively, both image and coil sensitivities can be jointly estimated from the same acquired data [18,19]. Uecker *et al*. [19] formulate MR image reconstruction as a nonlinear inverse problem and propose to solve equation (2.1) using the iteratively regularized Gauss Newton method (IRGNM). This method linearizes the nonlinear inverse problem at each Gauss Newton step k , and estimates the update δm≔(δx,δc) for the pair $m^{k}≔\left(x^{k},c^{k}\right)$ by minimizing a regularized least-squares functional for the linearized sub-problem:


(2.3) $$\begin{array}{rl} \min_{\delta m} & \frac{1}{2}\|F^{\prime }(m^{k})\delta m\;+\;F(m^{k})\;-\;y{\|}^{2}\\ & \;+\;\beta^{k}W(c^{k}\;+\;\delta c)\;+\;\alpha^{k}R(x^{k}\;+\;\delta x). \end{array}$$

Here, $W(c)=\|w⊙Fc{\|}^{2}$ is a penalty on the high Fourier coefficients of the coil sensitivities and R(x) is a regularization term on the image x , e.g. $\ell_{2}$ -norm [19], $\ell_{1}$ -sparsity in the wavelet domain [20] or total variation [21]; $\alpha_{k}$ and $\beta_{k}$ decrease at each iteration step.

### (b) Learned priors as regularization

Learned prior knowledge can be used for regularization in image reconstruction. Generative priors, such as variational autoencoder, autoregressive models (e.g. PixelCNN) and diffusion models, are used to incorporate empirical knowledge about images into iterative optimization algorithms. We want to use generative priors directly as a drop-in replacement for conventional priors in existing image reconstruction algorithms, which are often based on proximal methods.

The proximal operator for the log-prior log⁡p(x) is defined as


(2.4) $${\mathrm{prox}}_{t}(z)\;=\;\underset{x}{\arg\min}\;\frac{1}{2t}\|x-z{\|}^{2}+\log p(x).$$

When the mapping above is not analytically computable, the proximal operator could be approximated by minimizing equation (2.4) using gradient descent. Note that the minimization problem for the proximal operator is the same as for a denoising problem for complex Gaussian noise [22]. Assuming some regularity of the prior and noise-like properties of the error during reconstruction, the gradient can be expected to always point in the same direction towards denoised images. Therefore, the optimality condition at the solution is approximately


(2.5) $$0\approx \frac{1}{t}(prox_{t}(z)-z)+\nabla_{x}\log p(x).$$

The solution is then equivalent to a single gradient-descent step with an arbitrary initial guess and unit step size,


$$\begin{array}{rl} {\mathrm{prox}}_{t}(z) & \approx z-t\nabla_{x}\log p(x), \end{array}$$

which simply yields a gradient-descent step for the log-prior in the overall algorithm. In this work, two types of log-priors are used, which are described below.

**PixelCNN prior**. This prior is formulated using a joint distribution over the elements of an image vector:


(2.6) $$\log p(x;NET(\hat{\Theta },x))=\log p(x^{\left(1\right)})\prod_{i=2}^{n^{2}}p(x^{\left(i\right)}|x^{\left(1\right)},..,x^{\left(i-1\right)}),$$

where the neural network $\mathrm{NET}(\hat{\Theta },x)$ predicts the distribution parameters of a mixture of logistic distributions that is used to describe every pixel and where the dependencies between the channels for the real and imaginary parts are described with nonlinear dependencies [11]. All these parameters used to probabilistically model the image are predicted by a causal network [23] that encodes the relationship between pixels as formulated in equation (2.6). The gradient of $\log\;p(x;\mathrm{NET}(\hat{\Theta },x))$ with respect to x can be computed by backpropagation through the neural network.

**Probabilistic diffusion prior**. The diffusion probabilistic model proposed in [24] is constructed with a forward Markovian process and a learned reverse process. The forward process is to gradually transfer a data distribution $q(x_{0})$ to a smoother known distribution $q(x_{N})$ , e.g. a Gaussian distribution, by adding noise to data points. The reverse process is to undo this forward process with learned reverse transitions, which are described as


(2.7) $$\begin{array}{rl} p_{\theta }(x_{i-1}|x_{i}) & =CN(x_{i-1}|\mu_{\theta }(x_{i},i),\tau_{i}^{2}I), \end{array}$$

where $\tau_{i}$ can be computed from the noise scales $\sigma_{i}$ at each step and $\mu_{\theta }(x_{i},i)$ can be understood as a denoised image based on the smoothed prior at each noise level. Instead of learning this distribution directly, the gradient of the log-prior is learned for all noise scales:


(2.8) $$\begin{array}{rl} \nabla_{x_{i}}\log p_{\theta }\left(x_{i-1}|x_{i}\right) & =\frac{1}{\tau_{i}^{2}}\left(\sigma_{i}^{2}-\sigma_{i-1}^{2}\right)s_{\theta }\left(x_{i-1},i\right), \end{array}$$

where $s_{\theta }(x_{i},i)$ is a trained score network [25], which is computationally efficient because it avoids backpropagation. We refer the reader to [12] for details about this method. The reverse transitions start with $\sigma_{\text{max}}$ and end at a $\sigma_{\text{min}}\approx 0$ . In this work, we use $\sigma_{\text{max}}=0.3$ and $\sigma_{\text{min}}=0.01$ , and


(2.9) $$\sigma_{i}=\sigma_{\text{min}}+(\sigma_{\text{max}}-\sigma_{\text{min}})\cdot \log(1+(1-i/N)\cdot (e-1)).$$

Here, e is the Euler’s constant, i is bounded to the iteration step in the optimization algorithm and N is the number of noise scales that corresponds to the total number of iterations.

For linear reconstruction, the two regularization terms can be directly plugged into proximal optimization algorithms available in image reconstruction frameworks, such as the fast iterative shrinkage-thresholding method (FISTA) [26], or the alternating direction methods of multipliers [27].

Similarly, for nonlinear inverse problems, we can apply the regularization terms to the linearized sub-problem in equation (2.3). In nonlinear reconstruction, the image content x is usually smooth at early Gauss Newton steps and the distribution of x is far from the learned empirical distribution. Correspondingly, in this work, the Gauss Newton optimization is split into two stages. In the first stage, an $\ell_{2}$ -norm regularization is applied, and the method of conjugate gradients is used to minimize equation (2.3). In the second stage, i.e. the later Gauss-Newton steps, FISTA is utilized with the proximal operators. The entire algorithm is outlined in algorithm 1.

**Figure:**
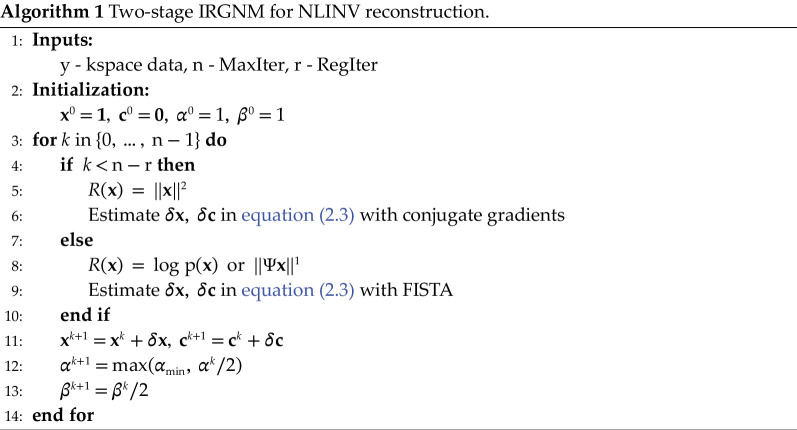


## 3. Methods

In this section, we first describe how we implemented the proposed workflow, as shown in figure 1, for extracting prior knowledge from an image dataset and then how we use the learned prior for regularization in image reconstruction. We detail how we evaluated the performance of the priors used for image regularization in different settings.

### (a) Preprocessing of the training dataset

As for training data, we use human brain images from the Autism Brain Imaging Data Exchange (ABIDE) [28,29]. After we downloaded the dataset, which comes in three-dimensional volumes in NII^1^ format, we performed the following steps to preprocess it.

(i) Load each three-dimensional volume and resample it with the conform function of NiBabel [30] to make its axial plane have a size of 256 × 256.
(ii) Split the volume into two-dimensional image slices that are oriented in the axial plane.
(iii) Add background Gaussian noise ( μ=0.003,σ=5 ) to all slices as it is usually present in MR images, and then normalize every slice by dividing its maximum pixel value.
(iv) Crop a 30 × 30 patch from the xy-corner of each normalized slice and compute the mean μ and standard deviation σ over all pixels of the patch.
(v) Exclude slices (without image content) from phase augmentation when mean μ<0.04 and standard deviation σ<0.0061 .

### (b) Phase augmentation

ABIDE images are provided solely as magnitude images without phase information. The magnitude of an MR image is determined by the proton density, relaxation effects and receive fields, while the phase is affected by the phase of the receive field, inhomogeneities of the static field, eddy currents and chemical shift. Phase augmentation can be used to add a phase to obtain more realistic complex-valued images. Here, we describe a procedure to obtain new samples with phase information from magnitude images using a previously trained prior for complex-valued images. The method is based on previous research [24] for the sampling of a posterior. Given the likelihood term of the magnitude p(m|x) and a prior for complex-valued images p(x) , the posterior of the complex image is proportional to p(x|m)∝p(x)⋅p(m|x) , where


$$\bar{m}=\sqrt{x_{r}^{2}+x_{i}^{2}},$$

and $x_{r}$ and $x_{i}$ are the real and imaginary parts, respectively. The likelihood term for a given magnitude image m is $p(m|x)=\delta (m-\sqrt{x_{r}^{2}+x_{i}^{2}})$ . To be able to apply gradient-based methods, we approximate this with a narrow Gaussian distribution:


(3.1) $$p(m|x)\propto \exp(-\epsilon \|m-\sqrt{x_{r}^{2}+x_{i}^{2}}{\|}_{2}^{2}).$$

Specifically, we initialize samples with random complex Gaussian noise and then transfer them gradually to the distribution of complex images with learned transition kernels $p_{\theta }(x_{n}|x_{n+1})$ . We run unadjusted Langevin iterations sequentially at each intermediate distribution:


(3.2) $$x_{n}^{k+1}\leftarrow x_{n}^{k}+\frac{\gamma }{2}\nabla_{x}\log p_{\theta }(x_{n}^{k}|x_{n+1}^{K})+\frac{\gamma }{2}\nabla_{x}\log p(m|x_{n}^{k})+\sqrt{\gamma }z.$$

Here, z is complex Gaussian noise, which introduces random fluctuations and γ controls the step size of the Langevin algorithm. The sampling algorithm was implemented with TensorFlow (TF) and used with the pre-trained generative model ${\mathrm{NET}}_{1}$ from [12], which was trained on the small dataset from [11]. For each magnitude image five complex images were generated.

### (c) Training of priors

In total, we trained six priors in this work. The PixelCNN priors, P_SC_ and P_SM_, were trained on the small brain image dataset used in [11] using complex and magnitude images, respectively. P_LM_ and P_LC_ were trained on a subset of the preprocessed ABIDE dataset corresponding to 500 volumes and the corresponding phase-augmented complex images, respectively. We also trained one diffusion prior, score matching with Langevin dynamics (SMLD) (D_SC_) with phase-augmented images using the full ABIDE dataset with 1206 volumes.

During the training, images were normalized to have a maximum magnitude of one and then subjected to random mirroring, flipping and rotation prior to being fed into the neural network. Complex images were fed as two-channel maps (i.e. real and imaginary), and magnitude images were fed as single-channel maps. The networks used for PixelCNN and the diffusion models were implemented with TF and the optimizer ADAM was used for all training tasks, which was performed using multi-GPU systems using different GPUs (Nvidia Corporation, Santa Clara, CA, USA). More information about priors and training is listed in table 1.

**Table 1.** Datasets and computational resources used to train the six different priors used in this work; P_SC_ trained from a small dataset of complex-valued images will serve as a baseline for the other learned priors.

prior		model	phase	no. of images	MR contrasts	GPUs	parameters	time × epochs
P_SC_	(small, complex)	PixelCNN	preserved	1000	T_1_, T_2_, T_2_-FLAIR, T ${,}_{2}^{*}$	4 × A100, 80 G	approx. 22 M	approx. 40 s × 500
P_SM_	(small, magnitude)	PixelCNN	not available	1000	T_1_, T_2_, T_2_-FLAIR, T ${,}_{2}^{*}$	4 × V100, 32 G	approx. 22 M	approx. 144 s × 500
P_LM_	(large, magnitude)	PixelCNN	not available	23 078	MPRAGE	4 × A100, 80 G	approx. 22 M	approx. 748 s × 100
P_LC_	(large, complex)	PixelCNN	generated	23 078	MPRAGE	3 × A100, 80 G	approx. 22 M	approx. 1058 s × 100
D_SC_	(SMLD, complex)	diffusion	generated	79 058	MPRAGE	4 × A100, 80 G	approx. 8 M	approx. 2330 s × 50

**PixelCNN prior.** We trained this generative model by maximizing the probability of the joint distribution over all the pixels in the image using the discretized logistic mixture distribution loss proposed in [23].

**Diffusion prior.** There exist two types of diffusion models that are based on the denoising score matching method [31], namely, denoising SMLD [32] and denoising diffusion probabilistic models [33]. Both are unified in a common framework described in [25]. We train diffusion priors using SMLD with the same Refine-Net [34] architecture also used in [12]. The loss function used to train the score network $s_{\theta }(x_{i},i)$ is given by


(3.3) $$\theta^{*}=\underset{\theta }{\arg\min}\;\sum_{i}E_{x_{0}}E_{x_{i}|x_{0}}[\lambda_{i}\|s_{\theta }(x_{i},i)-\nabla_{x_{i}}\log p\left(x_{i}|x_{0}\right){\|}_{2}^{2}],$$

where $\lambda_{i}$ is the weighting function described in [25].

### (d) Experimental evaluation

In this section, we use the Berkeley Advanced Reconstruction Toolbox [35] to evaluate the trained priors using parallel imaging compressed sensing (PICS) and nonlinear inversion (NLINV). The corresponding commands have an option for loading an exported TF computation graph and using it for regularization. The exported graph was wrapped into a nonlinear operator and then used in the proximal mapping step [36]. For the linear reconstruction using PICS, the coil sensitivities are estimated with ESPIRiT [37]. In the nonlinear reconstruction using NLINV, Algorithm 1 was implemented with the nonlinear operator framework [36].

We then performed different experiments using PICS and NLINV for the six priors using different sampling patterns in comparison to zero-filled, $\ell_{2}$ , $\ell_{1}$ -wavelet reconstructions and coil-combined images from fully sampled data. Here, we used a T_1_-weighted k-space (a common MR image contrast) from the test dataset used in [11]. In addition, we performed a study using quantitative image quality metrics using three-dimensional magnetization prepared rapid gradient echo imaging (MPRAGE) data to evaluate the effect of the size of the training dataset, and performed an evaluation study with human readers for six fully sampled three-dimensional TurboFLASH datasets as described below.

**The influence of phase maps**. We performed retrospective reconstruction using all six priors. Three types of undersampling patterns were used in this retrospective experiment, including fivefold acceleration along phase direction, two times and three times acceleration along frequency and phase direction, respectively, and 8.2 times undersampling using Poisson-disc sampling. While the acceleration along frequency-encoding direction is not realistic, we use it to explore how the priors handle different two-dimensional patterns. In a later experiment below, we then use three-dimensional k-space acquisitions where these sampling patterns are feasible.

**The influence of the size of dataset.** We performed the reconstruction using P_SC_ and P_LC_. The k-space data were acquired from the brain of a healthy volunteer using MPRAGE sequence on a 3T Siemens Skyra scanner (Siemens Healthineers, Erlangen, Germany) with 16-channel head coils. The protocol parameters were: TE = 2.45 ms, flip angle α=8 °, inversion time (TI) = 900 ms and TR = 2000 ms, 4/5 partial parallel Fourier imaging and twofold acceleration along one phase-encoding direction. This acquired three-dimensional volume has dimensions of 256 × 256 × 224 and an isotropic voxel size of 1 mm. We further undersampled the acquired three-dimensional k-space data two and three times along two phase-encoding directions with the central region of size 30 × 25 reserved. The reconstruction was performed slice-by-slice in a two-dimensional plane. To quantitatively assess the robustness of the priors, we computed peak signal-to-noise ration (PSNR) and structural similarity index (SSIM) for the P_LC_-regularized reconstruction from four and six times undersampled k-space against the P_LC_-regularized reconstruction from two times undersampled k-space and compared it with PSNR and SSIM for the $\ell_{2}$ -wavelet regularization and the prior P_SC_.

**Three-dimensional reconstruction quality.** For reconstruction of three-dimensional datasets, we chose the diffusion models, D_SC_, which is less computationally expensive than PixelCNN. The k-space data were acquired from six volunteers using a three-dimensional TurboFLASH sequence (TE = 3.3 ms, TR = 2250 ms, TI = 900 ms, flip angle α=9 °) using a 3T Siemens Prisma scanner and a 64-channel head-coil. These acquired three-dimensional volumes have dimensions of 256 × 256 × 176 and an isotropic size of 1 mm. We undersampled the acquired three-dimensional k-space data using a prospectively feasible Poisson-disc pattern with 8.2 times undersampling. When applying the prior during reconstruction, all slices in the axial plane are padded to a size of 256 × 256. Following this, the prior is applied on all slices in parallel and the computed gradient will be resized to the original image size. The reconstructed volumes were blindly evaluated by three clinicians with variable experience in neuroimaging (approx. 20, 10 and 5 years). In this evaluation study, $\ell_{1}$ -wavelet reconstructions and a reference reconstructed from fully sampled k-space data by coil-combination were included.. The grading scale used in this study ranged from 5 to 1, where a score of 5 represents ‘excellent’ image quality and a 1 denotes ‘bad’ image quality.

## 4. Results

**Figure 2.**
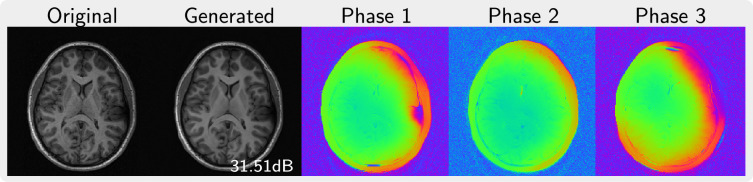
Human brain images. On the left, the original magnitude-only image is compared to the magnitude of a corresponding image generated using phase augmentation. On the right, the phase maps of three different generated images are shown.

Figure 2 shows an example of a magnitude image from the ABIDE dataset and the corresponding magnitude and phase maps of complex-valued images generated in the stage of phase augmentation. The magnitude part of the generated images stays very close to the original image but exhibits less noise. The phase of the generated images maps is smooth and looks realistic with some random variations introduced by the sampling algorithm.

### (a) The influence of phase maps

**Figure 3.**
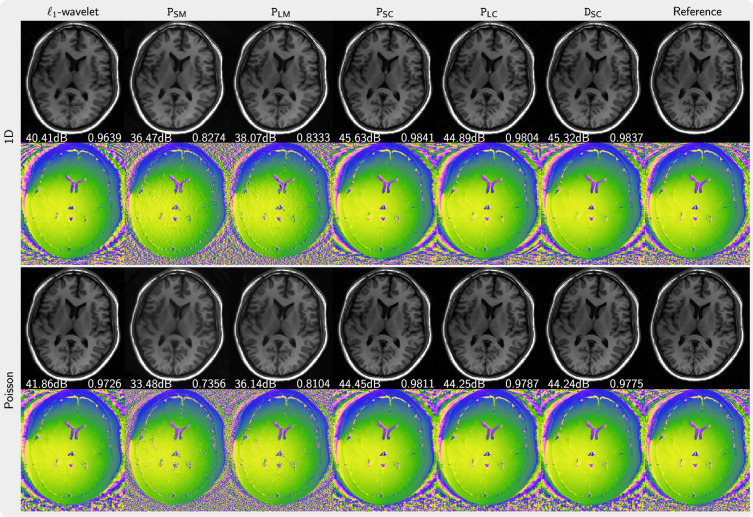
Comparison of images reconstructed using PICS using the priors P_SM_, P_LM_, P_SC_, P_LC_, D_SC_ in comparison to an $\ell_{1}$ -wavelet reconstruction and a reference (error maps are given in the electronic supplementary material). The top two rows (one-dimensional) present the results for fivefold acceleration along phase-encoding direction with 30 calibration lines. The bottom two rows (Poisson) show the results using a Poisson-disc acquisition of 8.2 times undersampling. PSNR and SSIM values are shown in white text.

**Figure 4.**
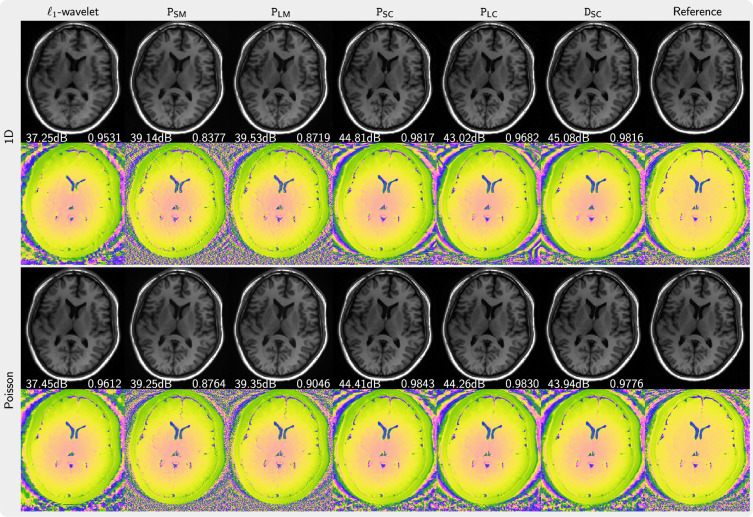
Comparison of images reconstructed using NLINV using the priors P_SM_, P_LM_, P_SC_, P_LC_, D_SC_ in comparison to an $\ell_{1}$ -wavelet reconstruction and a reference (error maps are given in the electronic supplementary material). The top two rows (one-dimensional) present the results for fivefold acceleration along the phase-encoding direction with 30 calibration lines. The bottom two rows (Poisson) show the results using a Poisson-disc acquisition of 8.2 times undersampling. PSNR and SSIM values are shown in white text.

**Figure 5.**
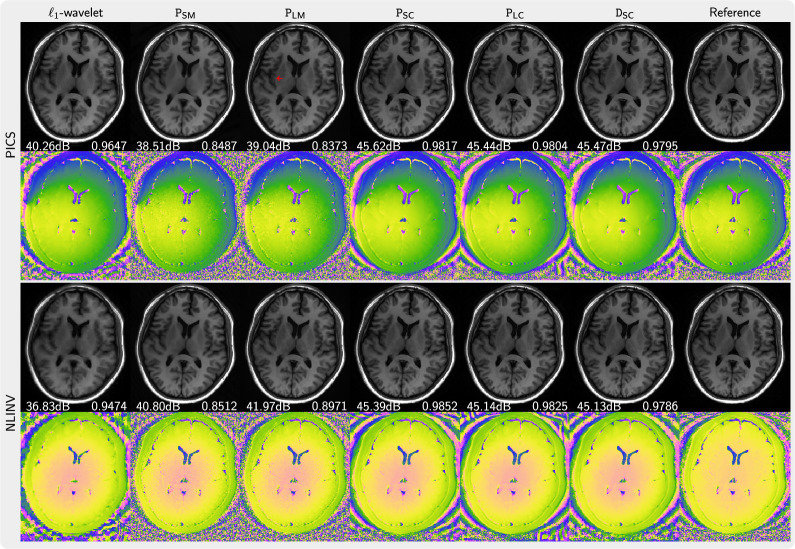
Comparison of images reconstructed using NLINV and PICS using the priors P_SM_, P_LM_, P_SC_, P_LC_, D_SC_ for a 2 × 3 sampling pattern in comparison to an $\ell_{1}$ -wavelet reconstruction and a reference (error maps are given in the electronic supplementary material); PSNR and SSIM values are shown in white text. Artefacts (red arrow) are introduced by the priors trained on magnitude images when using PICS.

Figure 3 presents the magnitude and phase of images that are reconstructed using PICS with priors trained from magnitude image, complex images with preserved phase and complex images generated using our phase augmentation method. While the priors P_SM_ and P_LM_ trained from magnitude images can remove folding artefacts introduced by undersampling, they exhibit over-smoothing of the magnitude as indicated by their lower PSNR and SSIM values and also demonstrate poor capabilities in denoising the phase. In contrast, the prior P_SC_ trained on complex-valued images performs much better. Furthermore, the priors P_LC_ and D_SC_ trained on phase-augmented images perform almost as well. Very similar results were obtained for NLINV as shown in figure 4. In figure 5, the k-space is sampled using 2 × 3 pattern. We observed artefacts (red arrow) introduced by the priors trained from magnitude-only images reconstructed with the PICS method, but not with the NLINV method. Under all investigated conditions, the priors trained on complex-valued images outperform the reconstruction with $\ell_{1}$ -wavelet regularization.

### (b) The influence of the size of dataset

**Figure 6.**
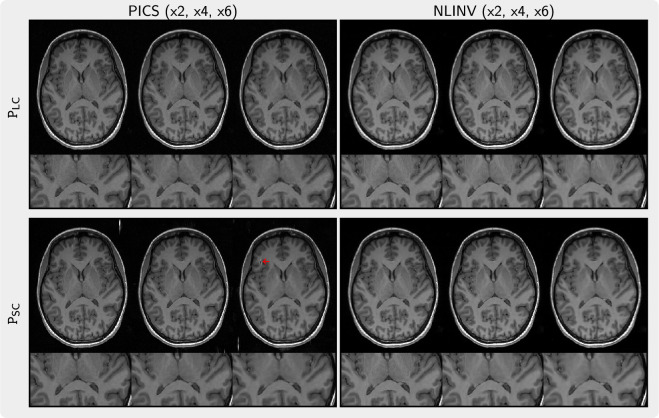
Comparison of images reconstructed with PICS (left) and NLINV (right) using priors P_LC_ (top) and P_SC_ (bottom) trained on small and large datasets. We observed artefacts (arrow) when using PICS with the prior P_SC_ trained on the small dataset. The images in each column are reconstructed from k-space undersampled with factors ranging from 2 to 6 (left to right).

Figure 6 presents the images regularized by the priors (P_SC_ and P_LC_) trained on small and large datasets, respectively. When using PICS with the prior P_SC_, artefacts become apparent in the background and in the brain, whereas no such artefacts are observed when applying the prior P_LC_. Furthermore, image details appear to be better preserved with high undersampling for the prior P_LC_.

**Figure 7.**
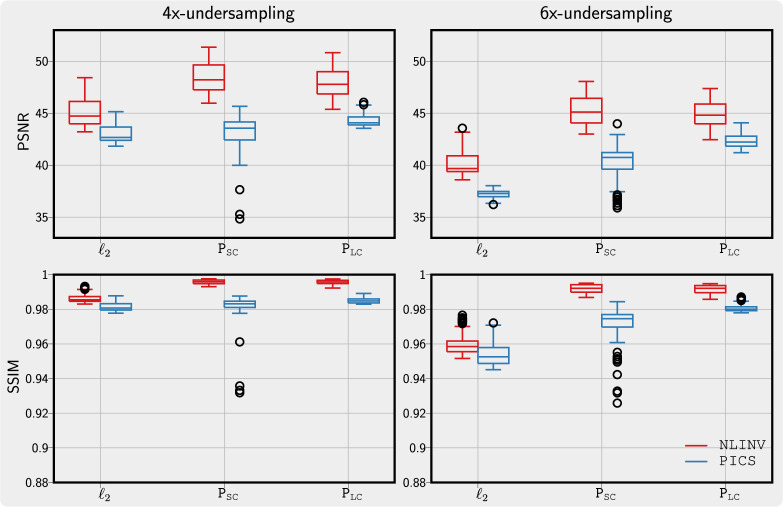
PSNR (top) and SSIM (bottom) metrics for images reconstructed with PICS (blue) and NLINV (red) when using $\ell_{2}$ -regularization and when using PixelCNN priors P_SC_ and P_LC_ for four times (left) and six times (right) undersampling relative to a reconstruction from two times undersampled k-space and using P_LC_. The PixelCNN trained on a larger dataset, P_LC_, shows fewer outliers compared with the one trained on a smaller dataset, P_SC_.

These observations can be confirmed quantitatively. We display the three sets of PSNR and SSIM metrics for $\ell_{2}$ , P_SC_ and P_LC_ with box plots in figure 7 for four times and six times undersampling relative to a reconstruction from two times undersampled k-space and using P_LC_. Here, the $\ell_{2}$ -regularization serves as a baseline reconstruction not influenced by the properties of a learned prior. For the P_LC_ prior learned from a large dataset, there are only a few outliers above the average when using PICS and NLINV. However, when using the P_SC_ prior learned from a small dataset, there are many outliers below the average, especially when the undersampling factor is high in the case of PICS.

### (c) Three-dimensional reconstruction using diffusion priors

**Figure 8.**
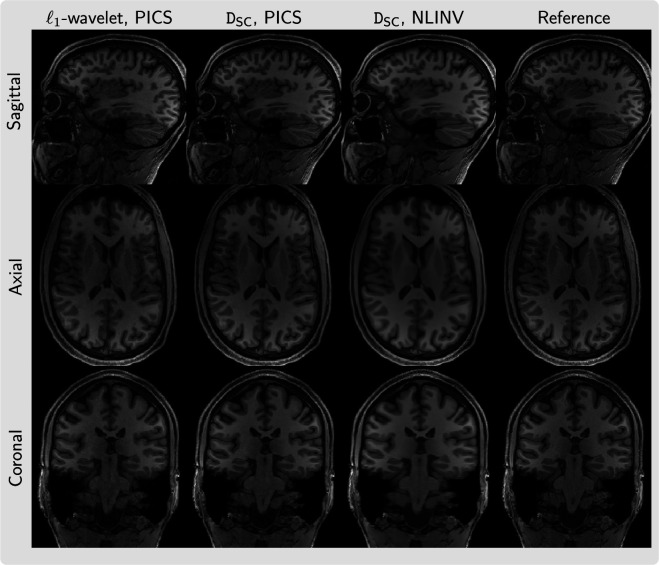
Slices in three orientations (sagittal, axial, coronal, from top to bottom) from a three-dimensional volume reconstructed using PICS from 8.2 times undersampled k-space with Poisson-disc sampling with $\ell_{1}$ -wavelet regularization and the diffusion prior D_SC_ and using NLINV with diffusion prior D_SC_ (from left to right).

As an example, figure 8 presents three slices in the sagittal, axial and coronal planes for a three-dimensional volume reconstructed using the diffusion prior D_SC_ using PICS and NLINV in comparison to $\ell_{1}$ -wavelet regularization and a reconstruction by coil combination of Fourier-transformed fully sampled k-space data. By visual inspection, the $\ell_{1}$ -regularized images appear to have reduced sharpness compared with the images regularized by the diffusion prior D_SC_, while also having more noise.

**Figure 9.**
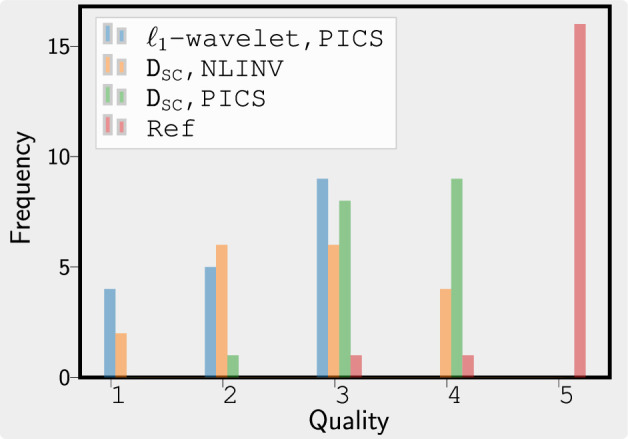
Blind evaluation by three clinicians of the volumes that are reconstructed with PICS using $\ell_{1}$ -wavelet regularization, PICS with diffusion prior D_SC_, NLINV with the diffusion prior and using coil combination of Fourier-transformed fully sampled k-space data. The grading scale ranges from 5 (excellent) to 1 (bad). There are 18 numbers indicating the image quality for each method, and the y-label ‘frequency’ means how often a certain scale of image quality appears in the evaluation.

Figure 9 shows the results from the evaluation by clinical readers. The diffusion prior D_SC_ outperforms $\ell_{1}$ -wavelet regularization leveraging the learned knowledge. Here, D_SC_ demonstrates better performance when using the PICS method compared with the NLINV method. With the relatively high acceleration factor of 8.2 used, none of the reconstructions matches the quality of the reference.

## 5. Discussion

A practical workflow was presented for extracting prior information from a set of magnitude-only images. It starts with the preparation of the training dataset, then followed by the generative modelling of complex-valued images and ends with the application of generative priors for regularization in image reconstruction. The effectiveness of the prior in boosting image quality was assessed by clinicians. Different aspects of this work are discussed below.

To exploit the information in magnitude-only images, the prediction of phase maps using a U-net was reported in [15]. Different from that, we prepared a training dataset of phase-augmented images through conditional generation using a complex diffusion prior that is first trained on a small dataset of complex-valued images. The prior trained on a large dataset of phase-augmented images exhibits high robustness, as shown in figure 7. The proposed approach will allow us to leverage the information in the large number of Digital Imaging and Communications in Medicine images already available in the archives of radiology departments.

In [38], the authors applied a conditional generative adversarial network (GAN) to produce phase maps based on magnitude images and then used them to synthesize k-space data. They reported a comparable performance to raw k-space data when the synthetic k-space data were utilized to train a variational network [6] for reconstruction. However, this still required running ESPIRiT on prior ground truth data from fastMRI [39] to obtain sensitivity maps for simulations. Zijlstra & While [40] expanded this idea by generating coil sensitivity phase maps based on magnitude images. This was achieved through a three-stage approach, involving the generation of low-resolution coil sensitivity phase maps based on magnitude images with a GAN, upsampling of low-resolution maps to high-resolution ones and transformation of the coil images to k-space data. Our work proposes a simple and less computationally intensive approach based on phase augmentation using a generic diffusion prior trained on complex-valued images. The advantage of this framework is that the learned prior is independent of k-space sampling patterns and coil sensitivities, and that it can be used as a regularization term in conventional reconstruction algorithms.

Training a prior is computationally expensive. For example, it took approximately 18 min to train P_LC_ per epoch through data parallelism using three A100 80G GPUs (Nvidia Corporation, Santa Clara, CA, USA). In contrast, the use of the prior in conventional reconstruction algorithms is computationally efficient. While previous reports indicate that up to 2000 evaluations of a diffusion prior are needed for reconstructing a single image [14], the number of evaluations required for a conventional linear reconstruction algorithm as used in this work is approximately 100. This work demonstrates how the curation of a dataset can significantly influence the performance and robustness of the prior in image reconstruction. The future work will involve a more systematic comparison of different generative models where the focus will be on the computational efficiency, robustness and reconstruction quality.

## 6. Conclusion

This work focuses on how to extract prior knowledge from existing magnitude-only image datasets using phase augmentation with generative models. The extracted prior knowledge is then applied as regularization in image reconstruction. The effectiveness of this approach in improving image quality is systematically evaluated across different settings. Our findings stress the importance of incorporating phase information and leveraging large datasets to raise the performance and reliability of generative priors for MRI reconstruction.

## Data Availability

In the spirit of reproducible research, the codes used to preprocess the dataset and to generate complex-valued images by phase augmentation using the diffusion prior, and the shell scripts for the reconstruction are made available in our repository: [11,12,36]. The Python library spreco used to train priors is available in this repository: [11,12,36,41]. Pre-trained models are made available at Zenodo: [42]. We refer readers to the webpage of our repository for additional information on the released materials. Supplementary material is available online

## Footnotes

^1^
The Neuroimaging Informatics Technology Initiative (NIfTI) is an open file format commonly used to store brain imaging data obtained using magnetic resonance imaging methods.
